# Immune reconstruction effectiveness of combination antiretroviral therapy for HIV-1 CRF01_AE cluster 1 and 2 infected individuals

**DOI:** 10.1080/22221751.2021.2017755

**Published:** 2021-12-28

**Authors:** Kang Li, Huanhuan Chen, Jianjun Li, Yi Feng, Guanghua Lan, Shujia Liang, Meiliang Liu, Abdur Rashid, Hui Xing, Zhiyong Shen, Yiming Shao

**Affiliations:** a Key Laboratory of Molecular Microbiology and Technology, Ministry of Education, College of Life Sciences, Nankai University, Tianjin, People’s Republic of China; b State Key Laboratory for Infectious Disease Prevention and Control, National Center for AIDS/STD Control and Prevention, Chinese Center for Disease Control and Prevention, Beijing, People’s Republic of China; c Guangxi Key Laboratory of Major Infectious Disease Prevention and Control and Biosafety Emergency Response, Guangxi Center for Disease Control and Prevention, Nanning, People’s Republic of China; d School of Public Health, Guangxi Medical University, Nanning, People’s Republic of China; e School of Medicine, Nankai University, Tianjin, People’s Republic of China

**Keywords:** HIV-1, genetic sub-cluster, antiretroviral therapy, immune reconstruction, coreceptor tropism

## Abstract

There are great disparities of the results in immune reconstruction (IR) of the HIV-1 infected patients during combined antiretroviral therapy (cART), due to both host polymorphisms and viral genetic subtypes. Identifying these factors and elucidating their impact on the IR could help to improve the efficacy. To study the factors influencing the IR, we conducted a 15-year retrospective cohort study of HIV-1 infected individuals under cART. The trend of CD4^+^ count changes was evaluated by the generalized estimating equations. Cox proportional model and propensity score matching were used to identify variables that affect the possibility of achieving IR. The tropism characteristics of virus were compared using the coreceptor binding model. In addition to baseline CD4^+^ counts and age implications, CRF01_AE cluster 1 was associated with a poorer probability of achieving IR than infection with cluster 2 (aHR, 1.39; 95%CI, 1.02-1.90) and other subtypes (aHR, 1.83; 95%CI, 1.31-2.56). The mean time from cART initiation to achieve IR was much longer in patients infected by CRF01_AE cluster 1 than other subtypes/sub-clusters (*P *< 0.001). In-depth analysis indicated that a higher proportion of CXCR4 viruses were found in CRF01_AE clusters 1 and 2 (*P *< 0.05), and showed tendency to favour CXCR4 binding to V3 signatures. This study indicated the immune restoration impairment found in patients were associated with HIV-1 CRF01_AE cluster 1, which was attributed to the high proportion of CXCR4-tropic viruses. To improve the effectiveness of cART, more efforts should be made in the early identification of HIV-1 subtype/sub-cluster and monitoring of virus phenotypes.

## Introduction

Human immunodeficiency virus type 1 (HIV-1) is characterized by extensive genetic diversity and evolved into various clades and circulating recombinant forms (CRFs) [1]. HIV-1 CRF01_AE was first discovered in 1989 among female sex workers in northern Thailand [2,3]. Phylogenetic analysis has indicated that the CRF01_AE virus originated in Central Africa in the 1970s and then transmitted to Thailand through sex workers in the 1980s [4,5]. Shortly afterward, it was demonstrated to be a recombinant HIV-1 virus circulating on a large scale in the world [6]. Since the HIV-1 CRF01_AE initial introduction to China in the 1990s, it has rapidly spread throughout the country and formed at least 7–8 genetic sub-cluster with various infection routes and geographic distributions characteristics [7–9]. HIV genetic diversity may affect immune recovery, disease progression, and response to antiretroviral treatment among infected patients [10–12]. Previous studies suggested that CRF01_AE was associated with faster disease progression from estimated seroconversion to acquired immune deficiency syndrome (AIDS) compared with non-CRF01_AE [10,13]. Another critical characteristic of the CRF01_AE strain was the high prevalence of CXCR4 (X4) co-receptor binding viruses [11,14].

The progression of the HIV patient’s immune reconstitution (IR) is associated with multiple factors, including baseline CD4^+^ cell, coreceptor usage, and viral genotype, but most previous studies have focused on subtype B [15–17]. With the diversity in HIV-1 virology clades and human genetics, understanding the progression of immune reconstitution between the major prevalent viral genotype and sub-cluster in China is vital to explore the interplay between viral clades and host immunity. Nevertheless, there are few studies on the relationship between immune reconstitution capability and HIV-1 circulating recombinant forms in China.

At present, some studies have demonstrated that CRF01_AE internal clusters had different virological characteristics and immunological responses, which patients infected with CRF01_AE cluster 4 (C4) had significantly lower baseline CD4^+^ cell counts and higher prevalence of X4 tropism in comparison with cluster 5 (C5) [8,18,19]. Unfortunately, the influence of these genetic clusters on IR progression to the combined antiretroviral therapy (cART) is still not fully understood. Especially the CRF01_AE cluster 1 (C1) and cluster 2 (C2) groups are widely distributed in the heterosexual infected population in Southeast Asia and the Southwestern border areas of China (e.g. Guangxi provinces) [20]. Although the Guangxi government has continued to strengthen the diagnosis and treatment of HIV patients in recent years, the mortality rate of HIV is still at a relatively high level. The HIV epidemic has brought unprecedented challenges to Guangxi and surrounding regions. Consequently, it is necessary to conduct a cohort study on the effect of antiviral treatment in HIV-1 patients in this region. Meanwhile, in this study, we also focused on investigating the effect of dominant HIV-1 subtypes/sub-clusters on CD4^+^ count recovery after cART in an observational cohort.

## Methods

### Participants

This study was a long-term cohort study involving adult HIV-positive patients (age ≥18 years) who received cART at Guangxi Center for Disease Prevention and Control between June 2003 and July 2018. Generally, the collected variables of the characteristics included baseline CD4^+^ count, CD4^+^ count during cART follow-up, gender, age at cART initiation, marital status, transmission category, treatment protocols, and various HIV-associated symptoms and complications (including tuberculosis infection, pneumonia, hepatitis, and meningitis). This study was reviewed and approved by the institutional review board of the National Center for AIDS/STD Control and Prevention, China CDC. Additionally, all study participants provided written informed consent at the time of sample collection.

### Genotype analysis and coreceptor tropism prediction

HIV-1 nucleic acid was extracted from 200 μl blood samples using Qiagen’s QIAamp Viral RNA Mini Kit according to the manufacturer’s instructions. Meanwhile, nested polymerase chain amplification (PCR) of the *env* (HXB2: 7002–7541 nt) region was performed according to the disclosed general method and all positive PCR products were directly sequenced [21,22]. Each step above has an appropriate negative control to prevent possible contamination during the experiment. HIV-1 subtypes were identified based on neighbor-joining tree analysis in comparison with general reference sequences. The phylogenetic tree was constructed by MEGA-X software with bootstrapping of 1000 replications [23]. Branches with bootstrap values above 90% were regarded as phylogenetic clusters [8,24,25]. Furthermore, the bootstrap values above 70% indicate that it was stable [9,26]. The HIV-1 *env* V3 loop region was used to predict the genotype of the co-receptor, which was the major determinant of viral tropism [27]. The Geno2pheno clonal model (https://coreceptor.geno2pheno.org/) was applied as a tool for judging viral tropism [28]. Based on our previous phenotypic verification studies, the CXCR4-tropism false positive rate (FPR) cut-off value was set below 2% [18].

### Definitions

According to the guidelines of the World Health Organization, China's current first-line combined Antiretroviral therapy plan includes stavudine (D4T) or tenofovir (TDF) or zidovudine (AZT) with lamivudine (3TC) and efavirenz (EFV) or nevirapine (NVP) [29,30]. From these cohorts, all of the individuals had good patient compliance and reached an undetectable HIV-1 RNA viral load during first-line cART treatment. Therefore, our primary analysis focused on patients’ IR capacity which was monitored based on cut-off points for CD4^+^ count. The IR was defined as twice successive CD4^+^ count greater than 500 cells/µL in follow-up tests after cART initiation, and poor IR was defined as the CD4^+^ count recovery persisting less than 500 cells/µL from cART initiation [31,32]. Moreover, the enzyme immunoassay (EIA) was carried out with the maximum HIV-1 restricted antigen affinity EIA kit, which identified the recent HIV-1 infection situation [18].

### Model building

The initial model of HIV *env*-V3 was a homology model calculated by SWISS-MODEL, selecting the Cryo-EM structure of *env* gp120 (PDB ID: 6NQD) as a template [33]. The structure of CXCR4 was also applied to the initial template for binding model construction [34]. The final model was analyzed by in PyMOL 2.5.0.

### Statistical analysis

We assessed characteristics of virus tropism in different genotypes, different CRF01_AE clusters and different baseline CD4^+^ count intervals with a Chi-squared test (for categorical data). Various methods were applied to assess changes in CD4^+^ cell count. The LOESS method was utilized to draw the trajectory of CD4^+^ count overtime after the start of cART. Meanwhile, we also used the generalized estimating equations modelling to examine longitudinal changes in CD4^+^ cell count growth and the influencing factors.

Kaplan-Meier analysis was used to estimate progression from cART initiation to achieving immune reconstruction (CD4^+^ count≥500 cells/µL) during the follow-up period, and the log-rank test was used to estimate statistical differences. We used both univariate and multivariate Cox proportional hazard models to evaluate the effects of HIV-1 genotypes on achieving immune reconstruction of HIV/AIDS patients, defined time as cART time, and tested the variables for proportional hazard (PH) assumption. In order to control for potential confounding, demographic characteristic factors were also included as controls in the adjusted models. In the data statistics applications, propensity score matching (PSM) is a commonly used statistical matching method which may reduce the bias due to confounding factors. For the 1:1 PSM in this study, the baseline variables that were significant different between IR group and non-IR group were marched using a caliper starting with at 0.02, and reducing the caliper width until all characteristics were matched, to avoid these variables will affect the estimated impact on the immune recovery capacity of HIV/AIDS individuals. Then, the chi-square test was performed to examine the validness of the propensity score model. Moreover, a multivariable conditional logistic regression was applied to estimate the independent effect of HIV-1 genotype on immune reconstruction. All analyses were performed using SPSS, version 23.0 and R, version 3.6.2.

## Result

### General characteristics of study participants at baseline

**Figure 1.**
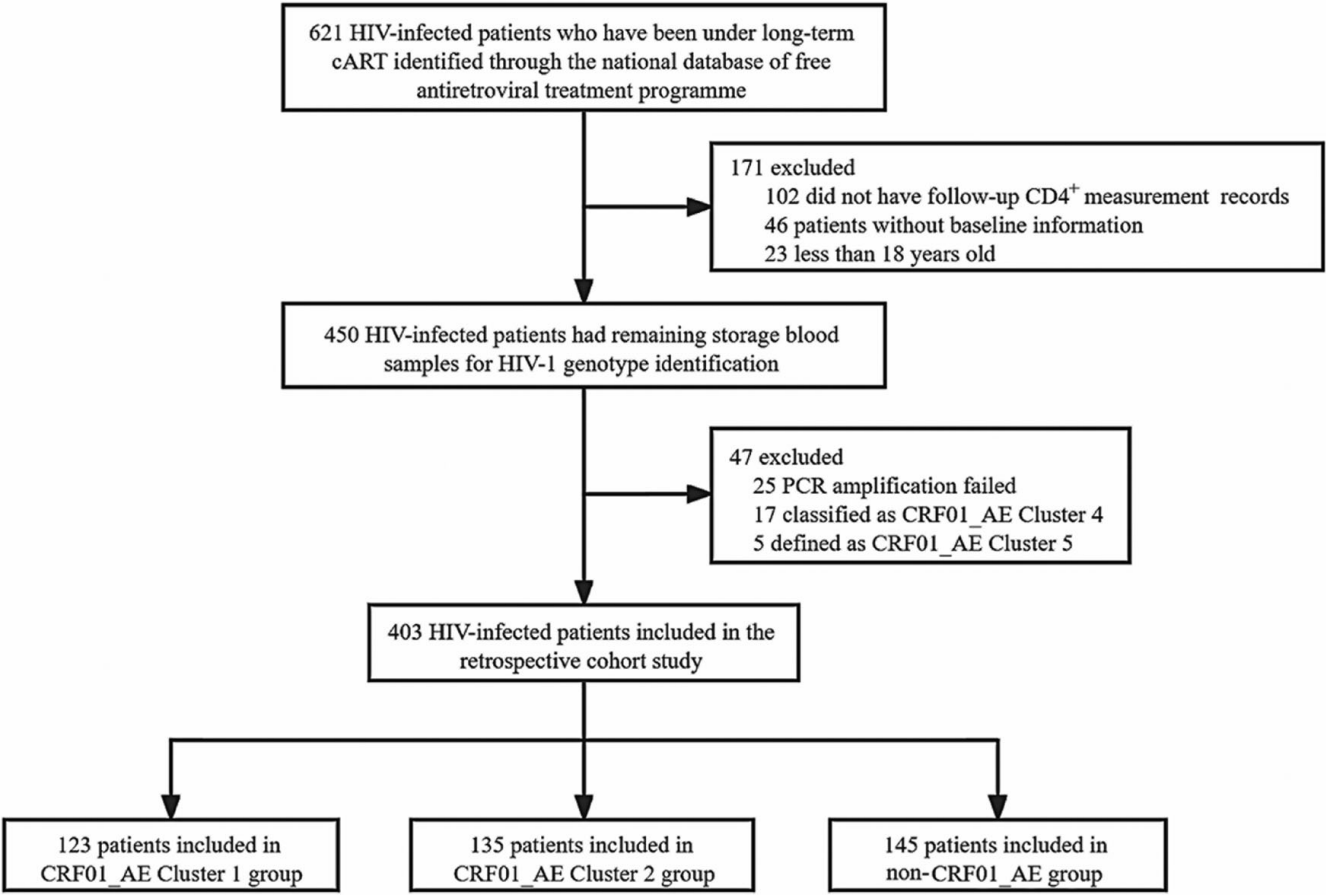
Flowchart of study participants filtration.

A total of 621 HIV patients who were undergoing cART between June 2003 and July 2018. Of these, 218 patients were excluded, including 102 missing follow-up CD4 + cell count records, 69 without baseline information or less than 18 years old, 25 PCR amplification failed, and 22 nucleotide sequences were classified as CRF01_AE other clusters. Ultimately, 403 patients met the eligibility criteria (Figure 1). Of these patients, the main genotype was CRF01_AE (123 with CRF01_AE cluster 1, 135 with CRF01_AE cluster 2) for 258 (64.0%), and other genotypes [i.e. subtype BC (103, 71.0%) and B’ (42, 29.0%)] which we classified as non-CRF01_AE groups, accounting for 145 (36.0%) (Figure S1). In the baseline demographic characteristics, 36.7% of patients were aged 30–39 years, 63.5% were male, 70.5% were married or cohabiting, 61.0% had a baseline CD4 + cell count <200 cells/μL, 76.4% infected through heterosexual contact and 17.1% male-to-male sexual contact (Table 1). Furthermore, the median follow-up time after the initial cART regimen was 411 weeks (IQR, 293−522 weeks). 34.7% (140/403) of participants received more than 10 years of cART-mediated viral suppression therapy.

**Table 1.** Baseline characteristics of study participants.

Variable	Total no. of patients (%)	CRF01_AE cluster1, n (%)	CRF01_AE cluster 2, n (%)	non-CRF01_AE, n (%)
				
Total	n = 403(100.0)	n = 123(30.5)	n = 135(33.5)	n = 145(36.0)
Age at cART initiation				
<30	124(30.8)	37(30.1)	33(24.4)	54(37.2)
30–39	148(36.7)	50(40.7)	51(37.8)	47(32.4)
40–49	72(17.9)	19(15.4)	29(21.5)	24(16.6)
50–59	37(9.2)	13(10.6)	16(11.9)	8(5.5)
≥60	22(5.4)	4(3.3)	6(4.4)	12(8.3)
Gender				
Male	256(63.5)	63(51.2)	90(66.7)	103(71.0)
Female	147(36.5)	60(48.8)	45(33.3)	42(29.0)
Baseline CD4^+^ cell count (cells/μL)				
≤200	246(61.0)	90(73.2)	99(73.3)	57(39.3)
201–299	89(22.1)	19(15.4)	21(15.6)	49(33.8)
≥300	68(16.9)	14(11.4)	15(11.1)	39(26.9)
Marital status				
Single, divorced or widowed	119(29.5)	30(24.4)	34(25.2)	55(37.9)
Married or cohabitation	284(70.5)	93(75.6)	101(74.8)	90(62.1)
Route of HIV transmission				
Heterosexual contact	308(76.4)	105(85.4)	111(82.2)	92(63.4)
Injecting drug use	20(5.0)	6(4.9)	7(5.2)	7(4.8)
Male-to-male sexual contact	69(17.1)	12(9.8)	17(12.6)	40(27.6)
Unknown	6(1.5)	0(0.0)	0(0.0)	6(4.1)
Recent infections				
Yes	87(21.6)	28(22.8)	28(20.7)	31(21.4)
No	316(78.4)	95(77.2)	107(79.3)	114(78.6)
Persistent fever or diarrhea				
Yes	111(27.5)	50(40.7)	39(28.9)	22(15.2)
No	292(72.5)	73(59.3)	96(71.1)	123(84.8)
With complication				
Yes	76(18.9)	40(32.5)	20(14.8)	16(11.0)
No	327(81.1)	83(67.5)	115(85.2)	129(89)

### Risk factors affecting the recovery of CD4 + cell count after cART

**Figure 2.**
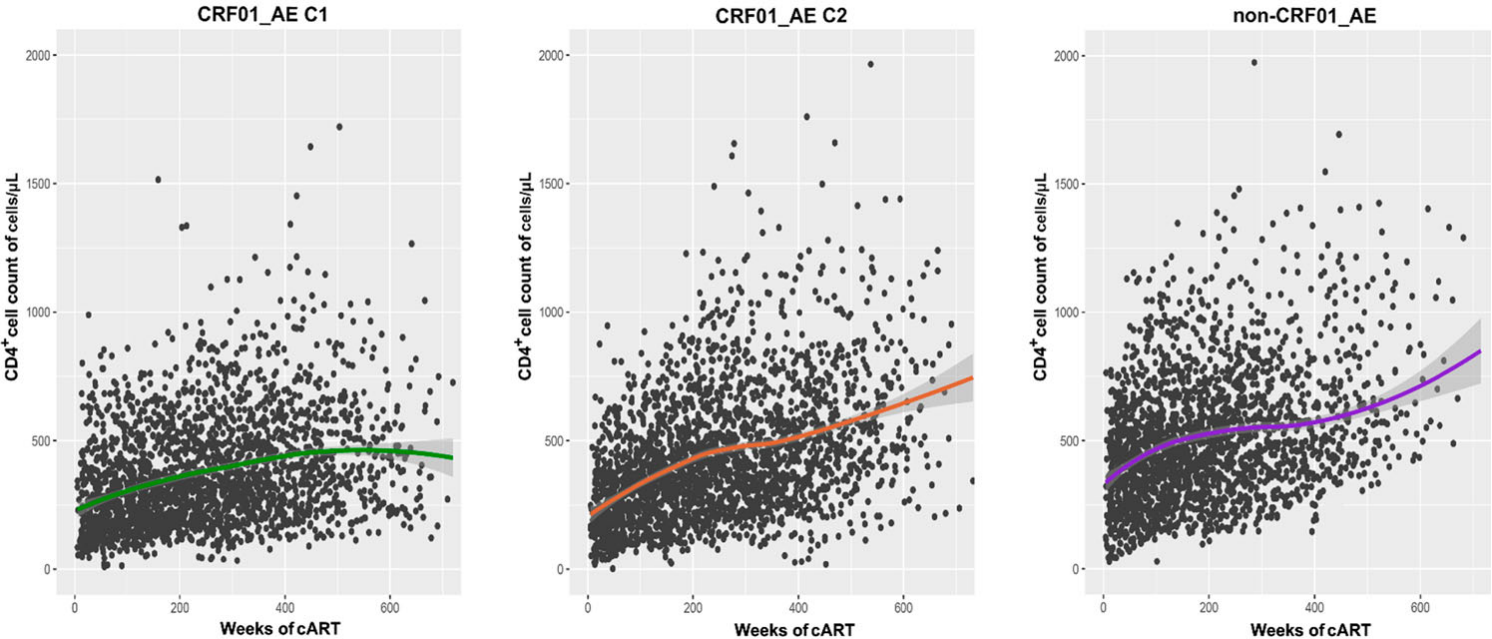
CD4^+^ cell count trajectory after cART initiation among different subtype/sub-cluster. Each point represents a participant's CD4^+^ cell count and the shaded area shows a 95% confidence interval. C1, cluster 1; C2, cluster 2.

Overall, during follow-up cART treatment, CRF01_AE infected patients had lower CD4^+^ cell count recovery than non-CRF01_AE group patients. In the two major CRF01_AE clusters, we observed that CD4^+^ cell count recovery ability after cART in cluster 1 patients was persistently weaker than in cluster 2 (Figure 2). After adjusting for the potential effects of other variables (e.g. gender, marital status, transmission route, fever, diarrhea and other complication) in a multivariable model, the lower CD4^+^ count after cART initiation was associated with HIV-1 genotype variables. Compared to those infected with CRF01_AE cluster 1, patients infected with CRF01_AE cluster 2 (54.27; 95% CI, 17.63-90.92) and non-CRF01_AE group (81.48; 95% CI, 41.44-121.52) had significant CD4^+^ cell higher increases. In addition, the high baseline CD4^+^ cell count and younger patients were associated with better recovery of CD4^+^ count (Table 2). Meanwhile, the same results were also shown in the recent infection group of the cohort. Similar results were seen using a univariate GEE model (Table S1).

**Table 2.** The analyze of factors related to CD4^+^ cell count growth trend after cART initiation using GEE model.

Attribute	Variable	All infections multivariate model		Recent groups multivariate model	
		Coefficient (95% Cl)	*P*-value	Coefficient (95% Cl)	*P*-value
Age at cART initiation	–	−2.18(−3.42–0.93)	<0.001	−0.81(−2.76-1.15)	0.419
Subtype	CRF01_AE cluster 1	1.00		1.00	
	CRF01_AE cluster 2	54.27(17.63-90.92)	0.004	135.2(71.62-198.79)	<0.001
	non-CRF01_AE	81.48(41.44-121.52)	<0.001	177.12(106.11-248.13)	<0.001
Baseline CD4^+^ cell count (cells/μL)	≥300	1.00		1.00	
	201–299	−142.46(−196.08–88.85)	<0.001	−98(−200.41-4.40)	0.061
	≤200	−261.7(−311.86–211.55)	<0.001	−246.46(−343.99–148.92)	<0.001

Note: Coefficient adjusted by sex, marital status, transmission route, persistent fever or diarrhea, with complication.

### Outcomes in propensity score matching (PSM) analysis

In the model with PSM, eight variables, including HIV transmission route, baseline CD4^+^ cell count, age, gender, marital status, recent infections, clinical symptoms, and complications, were properly matched, a final number of 192 participants (96 completed immune reconstruction and 96 uncompleted immune reconstruction) were included. There was no statistically significant difference in all matching variables between the two groups (Table S2). The conditional logistic regression analysis showed that the risk of immune reconstitution failure in the non-CRF01_AE group and CRF01_AE cluster 2 was lower than that of CRF01_AE cluster 1 [adjusted odds ratio (aOR) = 0.28, *P *=  0.003; aOR = 0.25, *P *< 0.001] (Table 3).

**Table 3.** Effect of subtype/sub-cluster on immune reconstruction among HIV patients receiving cART after propensity score matching.

Variable	Total, n	Immune-reconstruction failure, n (%)	OR (95% Cl)	*P*-value	AOR (95% Cl)	*P*-value
Subtype						
CRF01_AE cluster 1	63	42(66.7)	1.00	0.007	1.00	0.017
CRF01_AE cluster 2	81	36(44.4)	0.38(0.19-0.77)	0.007	0.39(0.17-0.87)	0.022
non-CRF01_AE	48	18(37,5)	0.28(0.12-0.65)	0.003	0.25(0.09-0.66)	0.005

Note: OR(odds ratio) were calculated by means of both univariate and multivariate logistic regression analysis; AOR (adjusted odds ratio) adjusted for age at diagnosis, sex, marital status, transmission route, baseline CD4 + cell count, persistent fever or diarrhea, with complication.

### Effects of HIV-1 genotype on the possibility of immune reconstruction after cART

**Figure 3.**
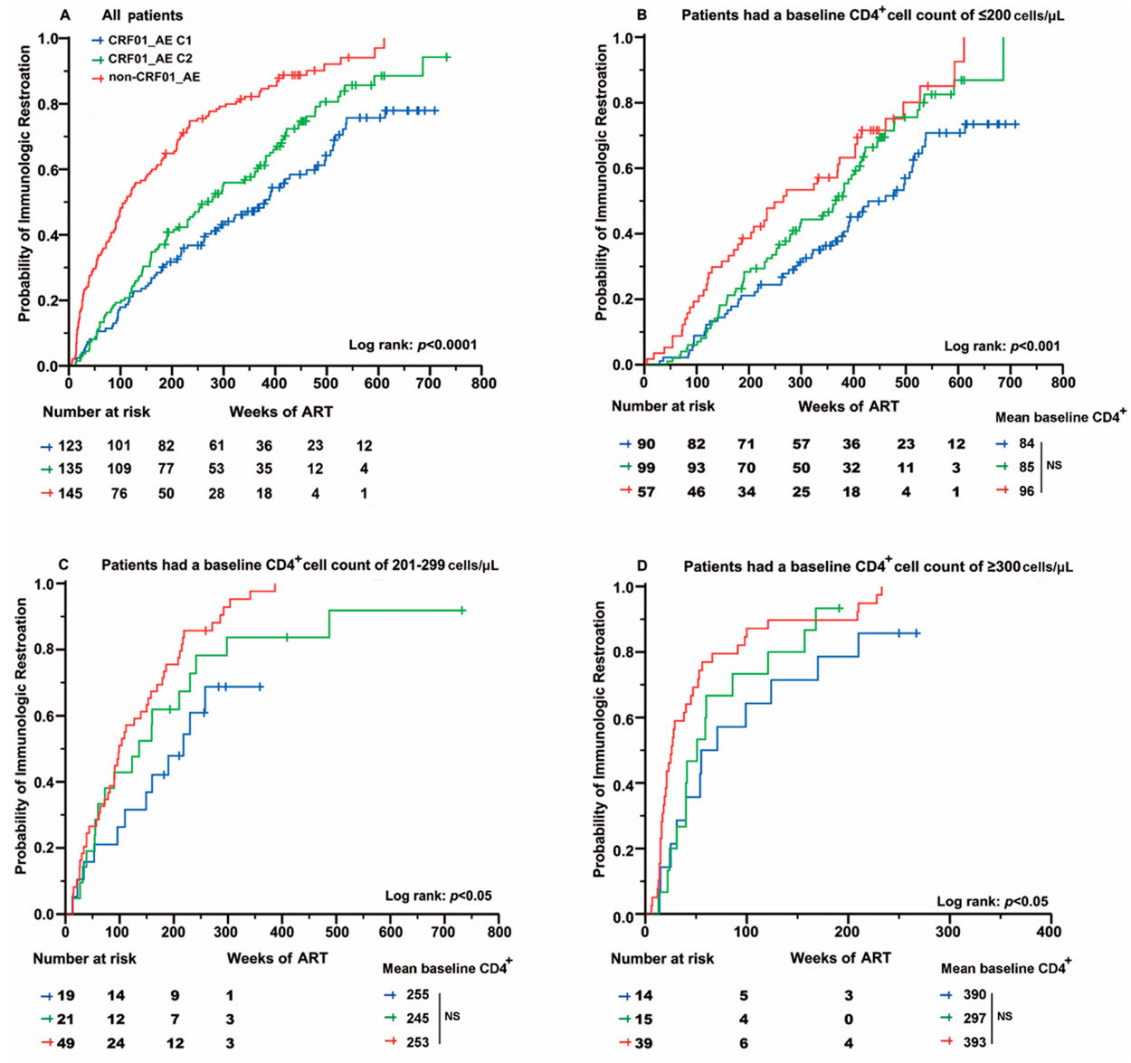
The possibility of each subtype/sub-cluster cART initiated to CD4^+^ cell count of 500 cell/uL immune reconstruction in different baseline CD4^+^ segmentation distribution. (A) All patients. (B) Patients had a baseline CD4^+^ cell count of ≤200 cells/μL. (C) Patients had a baseline CD4^+^ cell count of 201–299 cells/μL. (D) Patients had a baseline CD4^+^ cell count of ≥300 cells/μL. The initiation of cART was defined as time point zero. The statistical significance was measured by log-rank test. C1, cluster 1; C2, cluster 2.

Among patients confirming immune reconstruction, the meantime was longer among CRF01_AE cluster 1 (234 weeks) than CRF01_AE cluster 2 (223 weeks) and non-CRF01_AE subtypes (140 weeks) respectively (*P *< 0.001) (Figure 3A). The analyses also indicated that the immunological recovery progression was significantly different when participants were stratified by their subtype at almost all evaluation points. Cox regression analysis was used to determine the factors that affect the completion of immune reconstruction. As shown in Table 4, slower progression from cART initiation to achievement of immune reconstruction was associated with subtypes. Compared to CRF01_AE cluster 1 group, patients infected with CRF01_AE cluster 2 [adjusted hazard ratio (aHR), 1.51; 95% CI, 1.12-2.06] and non-CRF01_AE genotypes (aHR, 2.20; 95% CI, 1.60-3.03) had significantly higher probability of reaching a normal CD4^+^ cell count level. Similarly, in the recent infection group, the completion possibility of immune reconstruction in CRF01_AE cluster 1 was lower than that of other subtypes/sub-clusters. Analogous significant association results were seen in univariate analysis.

**Table 4.** Factors associated with time from cART initiation to immune reconstruction (CD4^+^ cell count > 500 cell/ul).

Variable	All infections multivariate model		Recent groups multivariate model	
	HR (95% CI)	aHR (95% CI)	HR (95% CI)	aHR (95% CI)
Subtype		** **	** **	** **
CRF01_AE cluster 1	1.00	1.00	1.00	1.00
CRF01_AE cluster 2	1.43(1.06-1.92)	1.51(1.17-2.06)	7.21(2.64-19.67)	8.25(2.86-23.75)
non-CRF01_AE	2.82(2.118-3.76)	2.20(1.31-2.56)	28.19(10.3-77.18)	18.83(5.01-70.82)
Baseline CD4 + cell count (cells/μL)				
≤200	1.00	1.00	1.00	1.00
201–299	3.24(2.44-4.3)	2.49(1.82-3.43)	7.36(3.46-15.67)	3.01(1.13-8.02)
≥300	8.76(6.34-12.11)	7.68(5.40-11.02)	44.28(16.8-116.74)	17.16(5.51-53.46)
Age at cART initiation	0.95(0.92-0.97)	0.96(0.94-0.99)	0.91(0.85-0.96)	0.97(0.91-1.00)

Note: HR (Hazard ratios) were calculated by means of both univariate and multivariate Cox regression analysis; aHR (adjusted hazard ratio) adjusted by sex, age at diagnosis, marital status, baseline CD4^+^ cell count, transmission route, persistent fever or diarrhea, with complication.

As the baseline CD4^+^ cell count was a major variable affecting immune reconstitution, we assessed the effect of the HIV-1 genotype on immune recovery in three subgroups of CD4^+^ cell count (≤200, 201-299, and ≥300 cells/μL). As shown in Figure 3(B-D), among all the subgroups, the probability of achieving a normal CD4^+^ cell count in the CRF01_AE cluster 1 was significantly lower than those in CRF01_AE cluster 2 and non-CRF01_AE group (*P *< 0.05). Furthermore, Cox regression analysis was used to determine the factors that affect the completion of immune reconstruction. After adjusting the baseline characteristic, the multivariate analysis demonstrated that the completion probability of CRF01_AE cluster 1 in the subtype group was significantly lower than others (*P *< 0.05) (Table S3).

### CXCR4 tropism was associated with lower CD4^+^ count recovery in specific subtype

**Figure 4.**
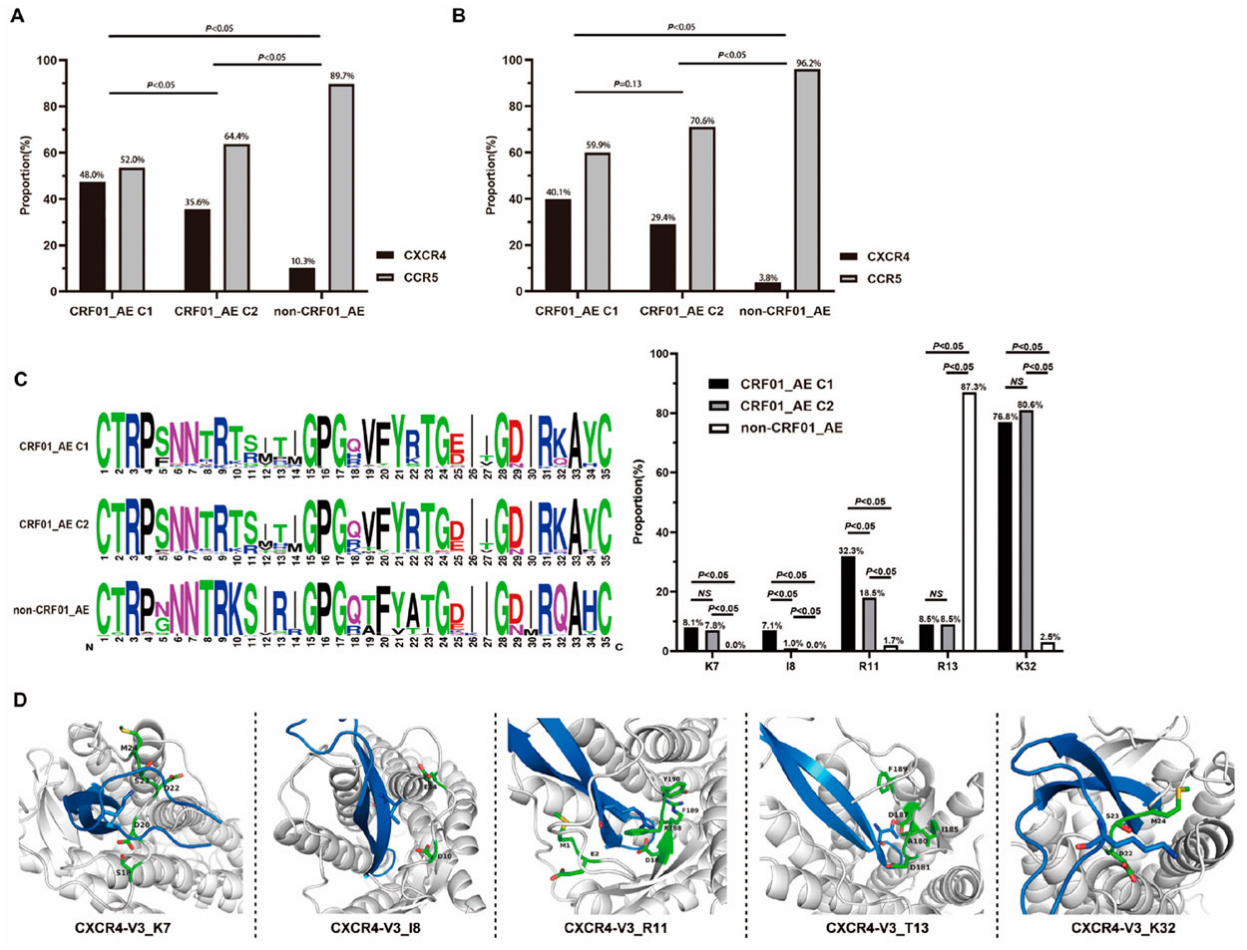
Distribution of tropisms in different subtype/sub-cluster and structural basis of the higher X4 utilization tendency. Distribution of tropisms based on viral subtype/sub-cluster (A). (B) Showed the distribution of tropisms in different subtypes/sub-clusters of groups that has achieved immune reconstitution. (C) Denoted the distribution of each V3 loop amino acid and the comparison of residues K7, T8, R11, R13 and K32 in different subtype/sub-cluster. The statistical significance was measured by ***χ***^2^ test. C1, cluster 1; C2, cluster 2. (D) Structural modelling for V3 positions 7, 8, 11, 13 and 32 in binding of coreceptors CXCR4 using the V3-docking model.

Compared to HIV patients infected with CCR5 (R5) tropism virus in the GEE and Cox models, patients infected with X4 tropism had significantly lower immune reconstitution probability and CD4 + cell count growth among patients under cART (all *P *< 0.05) (Tables S1 and S4). Moreover, the recent infection subgroup analysis of patients revealed the negative effects of X4 tropism on immune reconstitution probability. Therefore, we assessed the distribution of tropism in different subtypes and clusters. As shown in Figure 4(A-B), of all the 403 study participants, significantly higher propensity of X4 tropism was observed in all CRF01_AE (including cluster1 and cluster2) 107 (41.5%), than that in non-CRF01_AE (10.3%) (*P *< 0.001). Meanwhile, a higher proportion of X4 tropism was found in CRF01_AE cluster 1 (48.0%) compared with other subtypes. As X4 tropism was reported to be associated with poor CD4^+^ cell recovery [12,35,36], we analyzed the proportion of X4 tropism in different HIV-1 genotypes achieving CD4^+^ cell count to >500 cells/μL. It revealed that patients with CRF01_AE cluster 1 (40.1%) have higher X4 coreceptor tropism than with cluster 2 (29.4%, *P *= 0.13) and non-CRF01_AE group (18.3%, *P *< 0.001). To explore the underlying mechanism of X4 tropism usage tendency in different subtypes, we analyzed the proportion of these clade V3 amino acids sequences (Figure 4C). Compared to other subtypes, two highly conserved basic amino acids at positions R11 and K32 were observed in CRF01_AE cluster 1 and 2 V3 loop (*P *< 0.05). And the portion of CRF01_AE cluster 1 and 2 sequences miss the N-linked glycan site at the beginning of the V3 loop (V3 positions 6–8, HXB2 number 301–303), mainly by replacing the N/T at positions 7–8 with K/I. Interestingly, the percentage of I8 and R11 was also different in cluster 1 and cluster 2 (*P *< 0.05).

We applied the CXCR4-V3 complex models to study the role of HIV *env*-V3 positions 7, 8, 11, 13, and 32 in virus X4 tropism from a structural point of view (Figure 4D)[34]. In the CXCR4-V3 complex, positions 7, 11, and 32 of V3 were surrounded by negatively charged residues. The corresponding region in the ligand-binding pocket was negatively charged, which favours interaction with positively charged residue K/R in X4 sequences. Moreover, the residue K of V3 position 32 in clusters 1 and 2 may form salt bridges with CXCR4’s N-terminus which has more acidic residues than the N-terminus of CCR5. This explained why many CRF01_AE cluster 1 and 2X4 viruses sequences has more residue K/R at V3 position 7,11 and 32 than non-CRF01_AE group.

In addition, residue I8 in the V3 loop was surrounded by hydrophilic amino acids, indicating that a hydrophilic environment favours residue I8 more. In contrast, residue T13 in the X4 V3 loop surrounded hydrophobic amino acids (Figure 4D). Therefore, these two residues may be the key points for the higher trend of the X4 receptor use.

## Discussion

There are increasing evidences indicating that certain HIV clades or clusters have a faster disease progression. It is understandable considering HIV-1 is faster evolving virus jumped from animal to human about 100 years ago. The previous studies have demonstrated that CRF01_AE infected individuals were associated with fast HIV progression and advanced immunodeficiency [10,13,37]. However, there has been little exploration about the potential impacts of its internal sub-clusters on the capacity for CD4^+^ T-cell regeneration during long-term cART. According to the molecular epidemiological study, the CRF01_AE cluster 1 and 2 are dominant clusters circulating among the heterosexual population in Southeast Asia and Southern China [38]. The present study is a retrospective cohort study of individuals infected with non-CRF01_AE subtypes and two CRF01_AE genetic clusters for the first time. Our study demonstrated that CRF01_AE subtype (especially its cluster 1) is associated with a poorer ability to restore a normal peripheral CD4^+^ cell count. The reason may be that the ratio of X4 tropism in CRF01_AE cluster 1 was significantly higher [13,18,39]. The analysis of these subtypes is important as they emphasize that prior to antiretroviral therapy, certain subtypes of HIV/AIDS patients already have a substantial burden of immune recovery. Thus, the current treatment guidelines may be the inability to fully restore immunity to patients.

Previous studies have shown that most patients who can respond to viral suppression through antiviral therapy will continuously increase the number of peripheral CD4^+^ cells and eventually complete the individual's immune reconstruction within a certain period [31,40,41]. To further avoid potential bias caused by various factors, we conducted a sensitivity analysis in the multivariate model of HIV patients by stratifying the baseline CD4^+^ cell count and infection period, respectively. Meanwhile, we also carried out propensity score matching on the baseline variables. Ultimately, similar results to the overall cohort were obtained in all subgroups. The effectiveness of antiretroviral therapy will not only be affected by the HIV-1 subtypes, but also by its sub-cluster. Although baseline CD4^+^ cell count and age factors may also play a role [39,42,43], collectively, this study results provide strong evidence of the clinical impact that patients infected with HIV-1 CRF01_AE subtype and cluster 1 had poorer immune reconstruction ability than other subtypes. Even after 10 years of antiviral treatment, some CRF01_AE cluster 1 individuals cannot achieve the desired results. It suggested that local genotype and sub-clusters distribution should be considered in new guidelines for cART, such as CRF01_AE cluster 1. Moreover, for clinicians, the CD4^+^ cell count recovery of CRF01_AE and cluster 1 individual should be monitored more closely because these patients may develop AIDS more quickly than patients who are not infected with this genotype.

In terms of the distribution of viral tropism, in our cohort, we observed that the proportion of X4 tropism in CRF01_AE was much higher than that in other subtypes, which is an agreement with previous studies conducted in China that a high proportion of X4 tropism was reported in CRF01_AE subtype and demonstrated that X4 tropism is associated with lower CD4 counts and increased progression to immunosuppression [13,44]. Interestingly, we also observed imbalance within the sub-cluster, and the proportion of X4 tropism in cluster 1 patients was significantly higher than that in cluster 2. The study on the underlying mechanism showed that positions 11 and 32 of the V3 loop of the CRF01_AE cluster 1 and 2 had highly conserved basic amino acids, which were present in only about 1.7% and 2.5% of the other subtype viruses. Moreover, in the N-linked glycan site at the beginning of the V3 loop (V3 positions 6–8, HXB2 nos.301–303), the CRF01_AE cluster 1 and 2 sequences had lost the residue N/T at positions 7–8 site, which was replaced by Lysine and Isoleucine amino acids. Previous studies have shown that the preference for the CXCR4 co-receptor tropism was positively correlated with the K7, R11, R13 and K32 (HXB2 numbers K302, R306, R308 and K327) amino acid substitutions of V3 loop [18,45]. Therefore, this reveals a greater propensity to X4-using in CRF01_AE and its cluster 1. Most published reports indicated that X4 tropism could significantly impact faster CD4^+^ cell count decline and HIV-1 progression, leading to less effective cART [12,35,36]. Consequently, the significant difference in IR ability among the HIV patients with different genotypes could be attributed to the high proportion of X4 tropism in the CRF01_AE subtype and its cluster 1. Although this study was based on the viral tropism obtained by HIV-1 genotyping prediction, its accuracy has been verified by previous research in the same category [28,45].

Some research limitations should be noted. First, we only observed that CRF01_AE and CRF01_AE cluster 1 was related to poor IR and the viral tropism is an influential factor for low CD4^+^ cell count increase among patients. Still we cannot fundamentally elucidate its biological mechanism. It is necessary to further study the relationship between IR and other gene-encoded viral proteins. Second, although we have made great efforts to collect recent infection patients and potential factors, the group’s size and analysis model adjustment factors in our study were still limited. We believe that the sample size of infected participants should be increased, and more available clinical follow up data should be accessible.

In summary, our study had provided an up-to-date evaluation of the relationship between the subtypes/sub-clusters. and immune reconstruction progression after cART among diagnosed HIV patients. We determined that CRF01_AE and its cluster 1 were the critical risk factors associated with the lower CD4^+^ cell count gains ability and longer time required to complete the immune reconstruction. The underlying reason may be attributed to the significantly high proportion of X4-tropic virus in CRF01_AE and cluster 1. It also explained why the HIV epidemic in Guangxi was under greater pressure and patients were more difficult to treat. Nowadays, there are at least two changes have facilitated our capacity to do genotype and phenotype surveillance: the advancement of “next-generation” sequencing technology and the abundance of sequencing data generated by routine drug resistance monitoring. These progresses have improved the efficiency for such surveillance activities. Consequently, this study strongly emphasized the significance of timely genotyping and phenotyping surveillance to reduce the possibility of immune reconstruction failure and ensure patient’s quality life.

## Supplementary Material

Supplemental Material

## Data Availability

All data included in this study are available upon request by contact with the corresponding author.
