# Supplementary material for: Immune reconstruction effectiveness of combination antiretroviral therapy for HIV-1 CRF01_AE cluster 1 and 2 infected individuals
Source: Emerg Microbes Infect. 2021 Dec 28;11(1):158–67. doi: 10.1080/22221751.2021.2017755 (PMC8725829; doi:10.1080/22221751.2021.2017755)
Supplement: Supplemental Material [file TEMI_A_2017755_SM9826.docx]

**Immune reconstruction effectiveness of combination antiretroviral therapy for HIV-1 CRF01_AE cluster 1 and 2 infected individuals**

**Running Title:** Immune reconstruction was impaired in cART patients with HIV-1 CRF01_AE cluster 1 and 2 infection

Kang Li^1,2*^, Huanhuan Chen^3*^, Jianjun Li^3*^, Yi Feng^2^, Guanghua Lan^3^, Shujia Liang^3^, Meiliang Liu^4^, Abdur Rashid^5^, Hui Xing^2^, Zhiyong Shen^3#^, Yiming Shao^1,2,3#^

**Author Affiliations:**

^1^Key Laboratory of Molecular Microbiology and Technology, Ministry of Education, College of Life Sciences, Nankai University, Tianjin, China.

^2^State Key Laboratory for Infectious Disease Prevention and Control, National Center for AIDS/STD Control and Prevention, Chinese Center for Disease Control and Prevention, Beijing, China.

^3^Guangxi Key Laboratory of Major Infectious Disease Prevention and Control and Biosafety Emergency Response, Guangxi Center for Disease Control and Prevention, Nanning, China.

^4^School of Public Health, Guangxi Medical University, Nanning, Guangxi, China.

^5^School of Medicine, Nankai University, Tianjin, China.

**Corresponding author:**

Shenzhiyong

Guangxi Center for Disease Prevention and Control, Nanning 530000, Guangxi, China.

E-mail: shenzhiyong99999@sina.com

Yiming Shao,

Division of Research on Virology and Immunology National Center for AIDS/STD Control and Prevention China CDC, Beijing 102206, China.

Tel: +8610-58900981

E-mail: [yshao@bjmu.edu.cn](mailto:yshao@bjmu.edu.cn)

*These authors contributed equally to this article.

**Supplementary Information**


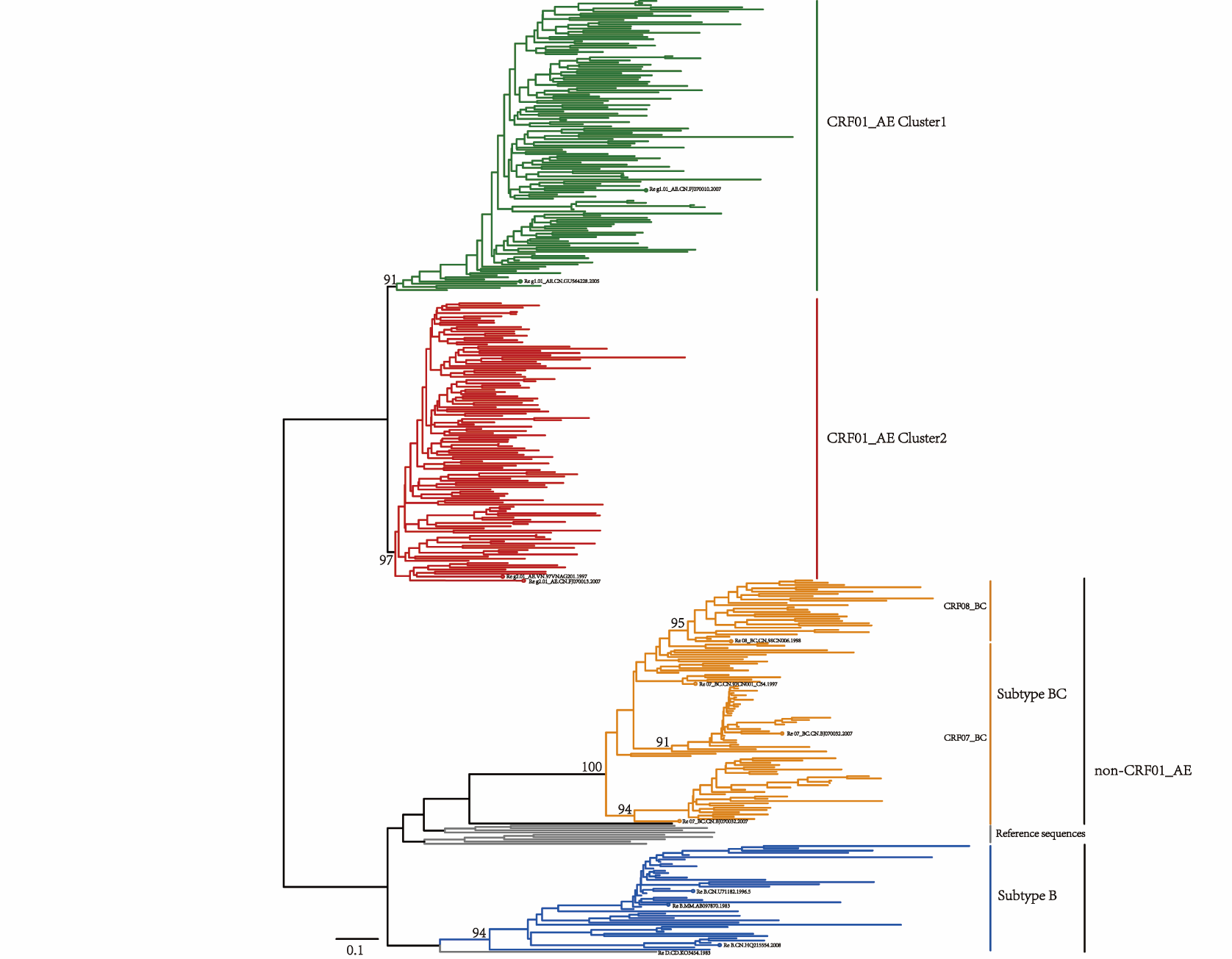


**Figure S1. Neighbor-joining phylogenetic tree of HIV-1 *env* sequences.**

Phylogenetic trees were constructed with study individual sequences and corresponding subtype references. The green and red lines represent CRF01_AE Cluster 1 and Cluster 2 respectively. Moreover, the subtype BC and B are labeled to dark yellow and blue lines.

| **Table S1 the analyze of factors related to CD4^+^ cell count growth trend after cART initiation using** **univariate GEE model** | | | | | | |
| --- | --- | --- | --- | --- | --- | --- |
| Attribute | Variable | All infections univariate model | |  | Recent groups univariate model | |
|  |  | Coefficient (95% Cl) | *P*-value |  | Coefficient (95% Cl) | *P*-value |
| Subtype | CRF01_AE cluster 1 | 1.00 |  |  | 1.00 |  |
|  | CRF01_AE cluster 2 | 54.69(41.24-68.13) | <0.001 |  | 104.19(78.6-129.79) | <0.001 |
|  | non-CRF01_AE | 143.93(130.35-157.51) | <0.001 |  | 288.48(263.23-313.74) | <0.001 |
| Baseline CD4 T cell count | ≥300 | 1.00 |  |  | 1.00 |  |
|  | 200-300 | -126.34(-145.16--107.52) | <0.001 |  | -163.13(-204.26--122) | <0.001 |
|  | ≤200 | -273.85(-289.82--257.87) | <0.001 |  | -375.52(-410.19--340.84) | <0.001 |
| Age at cART initiation | - | -4.02(-4.53--3.51) | <0.001 |  | -5.57(-6.62--4.52) | <0.001 |
| Gender | Female | 1.00 |  |  | 1.00 |  |
|  | Male | -38(-49.71--26.28) | <0.001 |  | -24.86(-50.85-1.14) | <0.001 |
| Marrital status | Married or cohabitation | 1.00 |  |  | 1.00 |  |
|  | Single, divorced or widowed | 61.82(49.33-74.31) | <0.001 |  | 149.68(126.02-173.34) | <0.001 |
| Transmission category | HET | 1.00 |  |  | 1.00 |  |
|  | IDUs | 50.9(24.74-77.06) | <0.001 |  | 298.3(228.96-367.65) | <0.001 |
|  | MSM | 58.65(43.45-73.84) | <0.001 |  | 115.34(81.09-149.59) | <0.001 |
|  | UN | 37.09(-15.52-89.71) | 0.167 |  | 63.84(-36-163.67) | 0.21 |
| Tropism | CXCR4 | 1.00 |  |  | 1.00 |  |
|  | CCR5 | 84.87(73.1-96.64) | <0.001 |  | 140.21(116.31-164.12) | <0.001 |
| Persistent Fever or diarrhea | No | 1.00 |  |  | 1.00 |  |
|  | Yes | -132.73(-146.29--119.17) | <0.001 |  | -163.7(-191.14--136.25) | <0.001 |
| With complication | No | 1.00 |  |  | 1.00 |  |
|  | Yes | -94.15(-105.54--82.76) | <0.001 |  | -79.22(-133.05--25.39) | <0.001 |
| Abbreviations: HET, heterosexual; MSM, men who have sex with men; IDUs, intravenous drug users; Unknown, data are not available; CI, confidence interval. | | | | | | |

| **Table S2 Comparison of the matching variables between two groups** | | | | |
| --- | --- | --- | --- | --- |
| Variable | Case | Controls | *χ2* | *P*-value |
|  |  |  |  |  |
|  | N (%) | N (%) |  |  |
| Total | 96(100.0) | 96(100.0) |  |  |
| Transmission category |  |  |  |  |
| Heterosexual contact | 79(82.3) | 75(78.1) | 3.14 | 0.37 |
| Injecting drug use | 5(5.2) | 5(5.2) |  |  |
| Male-to-male sexual contact | 12(12.5) | 13(13.5) |  |  |
| Unknown | 0(0.0) | 3(3.1) |  |  |
| Baseline CD4^+^ cell count (cells/μL） |  |  |  |  |
| ≤200 | 89(92.7) | 89(92.7) | 1.08 | 0.58 |
| 201-299 | 7(7.3) | 6(6.3) |  |  |
| ≥300 | 0(0.0) | 1(1.0) |  |  |
| Age |  |  |  |  |
| <30 | 19(19.8) | 17(17.7) | 1.86 | 0.76 |
| 30-39 | 42(43.8) | 44(45.8) |  |  |
| 40-49 | 15(15.6) | 20(20.8) |  |  |
| 50-59 | 13(13.5) | 11(11.5) |  |  |
| ≥60 | 7(7.3) | 4(4.2) |  |  |
| Gender |  |  |  |  |
| Male | 63(65.6) | 70(72.9) | 1.20 | 0.27 |
| Female | 33(34.4) | 26(27.1) |  |  |
| Marital status |  |  |  |  |
| Single, divorced or widowed | 23(24.0) | 27(28.1) | 0.43 | 0.51 |
| Married or cohabitation | 73(76.0) | 69(71.9) |  |  |
| Recent infections |  |  |  |  |
| Yes | 20(20.8) | 23(24.0) | 0.27 | 0.60 |
| No | 76(79.2) | 73(76.0) |  |  |
| Fever or diarrhea |  |  |  |  |
| Yes | 48(50.0) | 35(36.5) | 3.59 | 0.06 |
| No | 48(50.0) | 61(63.5) |  |  |
| With complication |  |  |  |  |
| Yes | 30(31.3) | 23(24.0) | 1.28 | 0.29 |
| No | 66(68.8) | 73(76.0) |  |  |

| **Table S3 Factors associated with time from cART initiation to immune recovery, group baseline CD4^+^ cell count** | | | | | | |
| --- | --- | --- | --- | --- | --- | --- |
| Group | Subtype/Cluster | Total no. of patients | univariate model | | multivariate model | |
|  |  |  | HR (95% CI) | *P*-value | aHR (95% CI) | *P*-value |
| CD4 cell count ≤200 cell/μL | CRF01_AE cluster 1 | 90 | 1.00 |  | 1.00 |  |
|  | CRF01_AE cluster 2 | 99 | 1.55(1.08-2.22) | 0.018 | 1.62(1.12-2.34) | 0.011 |
|  | non-CRF01_AE | 57 | 2.04(1.36-3.05) | 0.001 | 1.77(1.14-2.76) | 0.012 |
| CD4 cell count 201-299 cell/μL | CRF01_AE cluster 1 | 19 | 1.00 |  | 1.00 |  |
|  | CRF01_AE cluster 2 | 21 | 1.41(0.67-2.97) | 0.367 | 2.17(0.93-5.06) | 0.074 |
|  | non-CRF01_AE | 49 | 2.22(1.17-4.19) | 0.014 | 2.45(1.25-4.82) | 0.009 |
| CD4 cell count≥300 cell/μL | CRF01_AE cluster 1 | 14 | 1.00 |  | 1.00 |  |
|  | CRF01_AE cluster 2 | 15 | 1.47(0.67-3.23) | 0.335 | 1.58(0.67-3.7) | 0.295 |
|  | non-CRF01_AE | 39 | 2.16(1.12-4.15) | 0.021 | 3.47(1.43-8.46) | 0.006 |
| HR (Hazard ratios) were calculated by means of both univariate and multivariate Cox regression analysis; aHR: adjusted hazard ratio, adjusted for age at diagnosis, sex, marital status, transmission route, tropism, persistent fever or diarrhea, with complication. | | | | | | |

| **Table S4 Effects of different viral tropisms on immune recovery after cART among newly diagnosed HIV patients** | | | | | | | | | | | |
| --- | --- | --- | --- | --- | --- | --- | --- | --- | --- | --- | --- |
|  | CRF01_AE cluster 1 | |  | CRF01_AE cluster 2 | |  | non-CRF 01_AE | |  | Recent groups | |
| Tropism | HR (95% CI) | aHR (95% CI) |  | HR (95% CI) | aHR (95% CI) |  | HR (95% CI) | aHR (95% CI) |  | HR (95% CI) | aHR (95% CI) |
| CXCR4 | 1.00 | 1.00 |  | 1.00 | 1.00 |  | 1.00 | 1.00 |  | 1.00 | 1.00 |
| CCR5 | 1.70(1.08-2.69) | 2.61(1.74-3.91) |  | 1.54(1.02-2.30) | 1.63(1.06-2.50) |  | 8.68(3.51-21.46) | 6.61(2.64-16.56) |  | 4.34(2.33-8.07) | 4.85(2.86-8.21) |
| HR (Hazard ratios) were calculated by means of both univariate and multivariate Cox regression analysis; aHR: adjusted hazard ratio, aHR adjusted by sex, age at diagnosis, marital status, baseline CD4^+^ cell count, transmission route, persistent fever or diarrhea, with complication. | | | | | | | | | | | |
